# Downregulation of prenylation machinery genes in peripheral blood of patients with Parkinson’s disease: A pilot study

**DOI:** 10.1016/j.ibneur.2026.09.009

**Published:** 2026-09-12

**Authors:** Mohammad Jalal Tabatabaie, Solat Eslami, Atefe Abak, Soudeh Ghafouri-Fard

**Affiliations:** a Department of Medical Genetics, Shahid Beheshti University of Medical Sciences, Tehran, Iran; b Department of Medical Biotechnology, School of Medicine, Alborz University of Medical Sciences, Karaj, Iran; c Non-communicable Diseases Research Center, Alborz University of Medical Sciences, Karaj, Iran; d Phytochemistry Research Center, Shahid Beheshti University of Medical Sciences, Tehran, Iran

**Keywords:** Parkinson disease, FNTA, RABGGTA, RABGGTB, PGGT1B

## Abstract

**Background:**

Parkinson’s disease (PD) is a multifactorial neurodegenerative disorder, and peripheral blood-based biomarkers may help capture disease-related molecular changes. Because protein prenylation is essential for Rab-mediated intracellular trafficking, we investigated whether genes encoding key prenylation enzymes are dysregulated in PD.

**Methods:**

Peripheral blood samples were collected from patients with PD and healthy controls. Quantitative real-time PCR was performed for FNTA, FNTB, RABGGTA, RABGGTB, and PGGT1B, using B2M as the endogenous reference gene. Expression was analyzed with efficiency-adjusted ΔCt calculations, and group comparisons, regression analyses, correlation testing, and ROC analyses were performed using standard nonparametric and multivariable approaches.

**Results:**

Four genes, FNTA, FNTB, RABGGTB, and PGGT1B, were significantly downregulated in PD, whereas RABGGTA showed only a weak, non-significant decrease. Individual ROC analyses showed good-to-excellent diagnostic performance for FNTA, FNTB, RABGGTB, and PGGT1B, while RABGGTA performed poorly. A multigene logistic regression model combining FNTA, FNTB, RABGGTB, and PGGT1B yielded excellent discrimination between PD and controls (AUC = 0.9787), with 90.0% overall classification accuracy and satisfactory model calibration. Correlation analyses further suggested coordinated expression patterns among the studied genes, supporting a pathway-level disturbance.

**Conclusion:**

This pilot study identifies coordinated alterations in peripheral expression of prenylation-related genes in Parkinson’s disease. The findings support dysregulation of the protein prenylation machinery as a potentially informative molecular feature of Parkinson’s disease; however, the contribution of medication exposure and blood-cell composition could not be fully assessed, and independent external validation is required before clinical biomarker utility can be established.

## Introduction

Parkinson’s disease (PD) is the second most common progressive neurodegenerative disorder (Aarsland et al., 2021), characterized pathologically by the loss of dopaminergic neurons in the substantia nigra pars compacta and the accumulation of α-synuclein-containing Lewy bodies (Zhou et al., 2023). While the majority of PD cases are sporadic, mutations in several genes, including *SNCA*, *LRRK2*, *Parkin*, and *PINK1* have been implicated in monogenic forms of the disease (Cherian and Divya, 2020). Despite these advances, the molecular mechanisms underlying PD pathogenesis remain incompletely understood, and novel pathways contributing to disease onset or progression continue to be explored.

One emerging area of interest is protein prenylation, a post-translational modification involving the covalent attachment of either farnesyl or geranylgeranyl isoprenoid lipids to cysteine residues of target proteins (Palsuledesai and Distefano, 2015). This modification is essential for the proper membrane localization, trafficking, and function of numerous small GTPases, including Rab, Rho, and Ras families, which play critical roles in vesicular transport, cytoskeletal dynamics, and intracellular signaling (Hutagalung and Novick, 2011). In the context of PD, impaired vesicular trafficking (Hunn et al., 2015) and endolysosomal dysfunction (Kett and Dauer, 2016, Yahya et al., 2023) have been increasingly recognized as key pathogenic mechanisms. Rab proteins as central regulators of intracellular membrane trafficking (Bhuin and Roy, 2014) depend entirely on protein modifications for their activity, the enzymatic machinery responsible for this modification warrants further examination.

Protein prenylation process is mediated by three distinct heterodimeric enzymes: farnesyltransferase (FTase), geranylgeranyltransferase type I (GGTase-I), and geranylgeranyltransferase type II (GGTase-II), also known as Rab geranylgeranyltransferase (RabGGTase) (Marchwicka et al., 2022). The functional holoenzymes require specific subunits: FTase comprises FNTA (alpha subunit) and FNTB (beta subunit); GGTase-I comprises FNTA and PGGT1B; and RabGGTase comprises RABGGTA (alpha subunit) and RABGGTB (beta subunit) (Pinner et al., 2020). Despite the known prominence of prenylation in neuronal health, protein trafficking and synaptic plasticity (Hottman and Li, 2014), the expression levels of these prenyltransferase subunit genes have not been systematically assessed in PD.

To address this gap, the present study evaluated the expression of four key prenyltransferase subunit genes—*FNTA* (shared by FTase and GGTase-I), *PGGT1B* (beta subunit of GGTase-I), *RABGGTA*, and *RABGGTB* (alpha and beta subunits of RabGGTase)—in the context of PD. By characterizing the transcriptional profile of these critical enzymes, this investigation aims to provide initial insights into whether alterations in the prenylation machinery could contribute to trafficking defects and neurodegeneration in PD.

## Materials and methods

### Patients and controls

A total of 50 individuals with PD and 49 healthy volunteers took part in the study. PD participants were enrolled between 2020 and 2021 from Farshchian Hospital in Hamadan, Iran. The control group consisted of individuals attending routine health check-ups during 2025. PD diagnosis followed the criteria established by the International Parkinson and Movement Disorder Society (Goetz et al., 2004). Exclusion criteria included acute or chronic infections, cancerous conditions, or systemic diseases. Functional impairment, cognitive status, and disease severity and progression were evaluated using the Hoehn and Yahr (H&Y) staging, the Mini-Mental State Examination (MMSE), and the Unified Parkinson’s Disease Rating Scale (UPDRS), respectively (Ebersbach et al., 2006). The study was approved by the ethics committee of Shahid Beheshti University of Medical Sciences (IR.SBMU.MSP.REC.1404.443), and all participants—both PD patients and healthy controls—provided written informed consent.

### Expression assay

Total RNA was isolated from blood samples using an RNJia kit (Roje Technologies, Iran). Complementary DNA (cDNA) was then synthesized with a commercial cDNA synthesis kit (ADDBIO, South Korea). The expression levels of FNTA, RABGGTA, RABGGTB, and PGGT1B were measured using RealQ Plus 2x Master Mix (AMPLIQON, Denmark) and custom primers. PCR conditions and primer sequences matched those detailed in our earlier publication (Taheri et al., 2018). Quantitative real-time PCR (qPCR) was carried out for 45 cycles across all specimens. Primer-specific amplification efficiencies were calculated with LinRegPCR, which derives efficiency from the exponential phase of individual amplification curves and produces a mean efficiency value for each target. Relative transcript levels were determined using an efficiency-corrected ΔCt method, with B2M as the endogenous control; ΔCt was calculated as Ct[B2M] − Ct[target].

Transcripts that did not reach the fluorescence threshold within 45 cycles were labeled as non-detects (Ct > 45). To enable their inclusion in subsequent statistical analyses, these cases were conservatively assigned a Ct value of 46. Samples with undetermined B2M amplification or insufficient reference gene amplification were omitted from expression analyses. As a result, seven PD samples and two control samples were excluded before statistical evaluation. Where relevant, relative fold changes between groups were calculated using the 2^−ΔΔCt method to facilitate biological interpretation of expression differences.

### Statistical analysis

Statistical analyses were carried out using GraphPad Prism version 10.6.1 (GraphPad Software, La Jolla, CA, USA) and GenEx software (Multid Analyses AB, Gothenburg, Sweden). Normalized expression data for FNTA, FNTB, RABGGTA, RABGGTB, and PGGT1B were used in all subsequent analyses.

The normality of continuous variables was assessed with the Shapiro–Wilk test. Age differences between PD patients and healthy controls were examined using the Mann–Whitney *U* test, while sex distribution was compared via the chi-square test. Since normalized expression values deviated from a normal distribution, between-group comparisons for all target genes were conducted using the Mann–Whitney *U* test.

To explore whether expression differences were independently linked to disease status, multivariable linear regression models were built with gene expression level as the dependent variable and disease status, age, and sex as independent variables. Receiver operating characteristic (ROC) curve analysis was then performed to assess the ability of individual genes to discriminate between groups. Optimal cutoff values were determined using the Youden index, and the corresponding sensitivity, specificity, accuracy, positive predictive value (PPV), negative predictive value (NPV), and area under the ROC curve (AUC) were calculated.

To evaluate the combined diagnostic performance of FNTA, FNTB, RABGGTA, RABGGTB, and PGGT1B, multivariable logistic regression was performed, and the predicted probabilities from the model were subjected to ROC curve analysis. The diagnostic accuracy of the combined model was quantified by estimating the AUC. To assess potential optimism and overfitting of the multivariable logistic regression model, internal validation was performed using stratified 5-fold cross-validation. In each iteration, the model was fitted on the training subset and predicted probabilities were generated for the held-out subset. Out-of-fold predictions from all folds were pooled to estimate the cross-validated ROC AUC and classification accuracy. The apparent AUC obtained from the full dataset was reported separately from the cross-validated AUC. Cross-validation was considered an internal validation procedure and was not regarded as a substitute for external validation in an independent cohort.

Correlations among gene expression levels were evaluated using Spearman's rank correlation coefficient. Spearman's analysis was also applied to examine associations between gene expression and clinical variables, including age, sex, disease duration, H&Y stage, MMSE score, and UPDRS score. All statistical tests were two-tailed, and a p-value of less than 0.05 was deemed statistically significant.

## Results

### Participant characteristics

Table 1 presents the baseline characteristics of the study population. There were no significant differences between the PD patients and the healthy controls regarding age (p = 0.90) or sex distribution (p = 0.77). Most patients were in H&Y stages I–III and were undergoing treatment with L-DOPA-based regimens.

**Table 1:** General data of study participants.

**Study groups**	**Parameters**	**Values**		
Patients	Sex (number)	Male		37
		Female		13
	Age (Years, mean ± SD (range))	Male		69.64 ± 10.59 (47–89)
		Female		66.46 ± 12.6 (38–85)
	Disease duration (Years, mean ± SD (range))	Male		3.18 ± 3.65 (1–12)
		Female		5.38 ± 9.76 (1–36)
	MMSE (score, mean ± SD (range))	Male		22.83 ± 3.03 (17–29)
		Female		23.07 ± 2.49 (19–26)
	UPDRS (score, mean ± SD (range))	Male		23.9 ± 7.41 (13–41)
		Female		26.3 ± 9.43 (16–42)
	Hoehn & Yahr stage (Count)	I	Male	8
			Female	3
		II	Male	18
			Female	5
		III	Male	11
			Female	5
Controls	Sex (number)	Male		35
		Female		14
	Age (Years, mean ± SD (range))	Male		70.74 ± 8.76 (56–101)
		Female		69.64 ± 6.53 (61–83)

### Differential expression of FNTA, FNTB, RABGGTA, RABGGTB, and PGGT1B

**Fig. 1:**
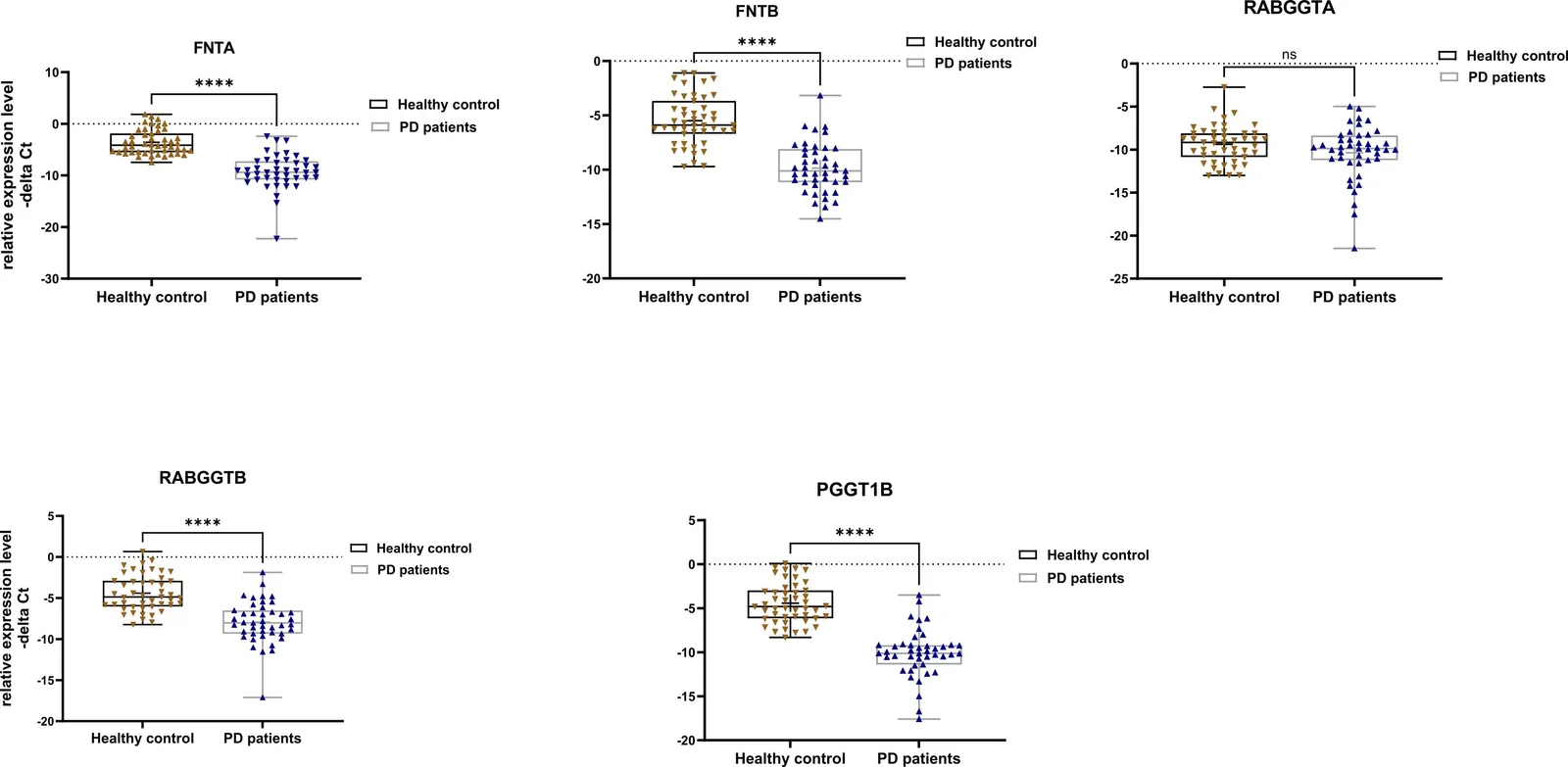
Differential expression of five protein coding genes FNTA, FNTB, RABGGTA, RABGGTB, and PGGT1B in peripheral blood samples from patients with Parkinson’s disease (PD) and healthy controls. Normalized expression values were calculated as Ct[B2M] − Ct[target] using B2M as the internal control and are displayed as box-and-whisker plots showing the median, mean, interquartile range, and minimum-to-maximum range. Between-group comparisons were performed using the Mann–Whitney *U* test. ^**^P < 0.01, ^**^P < 0.0001.

As shown in Fig. 1, normalized expression values (Ct[B2M] − Ct[target]) were consistently lower in the PD group than in controls, indicating reduced transcript abundance across the prenylation-related gene set. Between-group comparisons identified significant downregulation of FNTA, FNTB, RABGGTB, and PGGT1B, whereas RABGGTA showed a weaker reduction that did not appear to be as robust as the other four genes. Consistent with these findings, fold-change analysis (Table 2) showed marked decreases in PD relative to controls, with the strongest reductions observed for FNTA (0.026), PGGT1B (0.024), FNTB (0.048), and RABGGTB (0.08); RABGGTA demonstrated the least pronounced change (0.5).

**Table 2:** Fold changes in FNTA, FNTB, RABGGTA, RABGGTB, and PGGT1B expression in peripheral blood samples from patients with Parkinson’s disease compared with healthy controls. Values are shown as mean fold change with 95% confidence intervals.

Studied genes	Expression ratio (95% CI)	P value
FNTA	0.026 (0.011–0.048)	< 0.0001
FNTB	0.048 (0.024–0.094)	< 0.0001
RABGGTA	0.5 (0.23–1.11)	0.08
RABGGTB	0.08 (0.04–0.17)	< 0.0001
PGGT1B	0.024 (0.01–0.044)	< 0.0001

### Independent effects of disease status, age, and sex on target-gene expression

Multivariable linear regression showed that disease status was independently associated with lower expression of FNTA, FNTB, RABGGTB, and PGGT1B, whereas the association for RABGGTA did not reach statistical significance. Sex had no significant independent effect on any of the five genes. Age showed a small but significant positive association with FNTA expression only, while no age effect was detected for the other genes. Overall, the regression analysis confirmed that the observed expression differences were primarily driven by disease status rather than sex, with only a limited age effect confined to FNTA (Table 3).

**Table 3:** Multivariable linear regression analysis of FNTA, FNTB, RABGGTA, RABGGTB, and PGGT1B expression in patients with Parkinson’s disease and healthy controls: independent effects of disease status, age, and sex.

**Gene**	**Variable**	**Coefficient (β)**	**Standard Error**	**P-value**
FNTA	Disease effect	-5.55 (-6.74 to −4.36)	0.59	< 0.0001
	Gender effect	-0.66 (-1.98 to 0.65)	0.66	0.31
	Age effect	0.07 (0.008–0.13)	0.03	0.027
FNTB	Disease effect	-4.36 (-5.34 to −3.39)	0.49	< 0.0001
	Gender effect	-0.04 (-1.12 to 1.04)	0.54	0.94
	Age effect	0.0003 (-0.05 to 0.05)	0.02	0.98
RABGGTA	Disease effect	-1 (-2.16 to 0.14)	0.58	0.08
	Gender effect	-0.38 (-1.65 to 0.89)	0.64	0.55
	Age effect	-0.01 (-0.076 to 0.042)	0.03	0.58
RABGGTB	Disease effect	-3.46 (-4.47 to −2.45)	0.5	< 0.0001
	Gender effect	-0.39 (-1.51 to 0.71)	0.56	0.47
	Age effect	0.02 (-0.028 to 0.07)	0.02	0.36
PGGT1B	Disease effect	-5.67 (-6.73 to −4.6)	0.53	< 0.0001
	Gender effect	-0.16 (-1.34 to 1)	0.59	0.77
	Age effect	0.00006 (-0.055 to 0.055)	0.02	0.99

### Intergene correlation patterns among FNTA, FNTB, RABGGTA, RABGGTB, and PGGT1B

Spearman’s rank correlation analysis showed a coordinated expression pattern among the five prenylation-related genes in both study groups, with the strongest and most consistent positive correlations observed in healthy controls. In controls, FNTA showed strong correlations with FNTB, RABGGTB, and PGGT1B, while FNTB was also strongly correlated with RABGGTB and PGGT1B; RABGGTB and PGGT1B demonstrated the strongest pairwise association. In the PD group, these associations remained positive but were generally attenuated, with the clearest correlations observed between FNTA and RABGGTB, FNTA and FNTB, FNTA and PGGT1B, and RABGGTB and PGGT1B. By contrast, RABGGTA displayed little or no meaningful correlation with the other transcripts in either group, suggesting that its expression was less tightly coupled to the remaining genes in this pathway (Table 4).

**Table 4:** Spearman’s rank correlations among FNTA, FNTB, RABGGTA, RABGGTB, and PGGT1B expression levels in patients with Parkinson’s disease (n = 43) and healthy controls (n = 47).

	FNTB		RABGGTA		RABGGTB		PGGT1B	
	Control	PD	Control	PD	Control	PD	Control	PD
FNTA	0.84^b^	0.36^a^	0.02	0.12	0.84^b^	0.55^b^	0.83^b^	0.36^a^
FNTB			-0.09	0.08	0.79^b^	0.25	0.87^b^	0.07
RABGGTA					0.1	- 0.01	-0.004	0.09
RABGGTB							0.86^b^	0.67^b^

a *p*-Value at a significance level of *p* < 0.05.

b *p*-Value at a significance level of *p* < 0.001.

### Diagnostic performance of FNTA, FNTB, RABGGTA, RABGGTB, and PGGT1B in distinguishing PD from controls

**Fig. 2:**
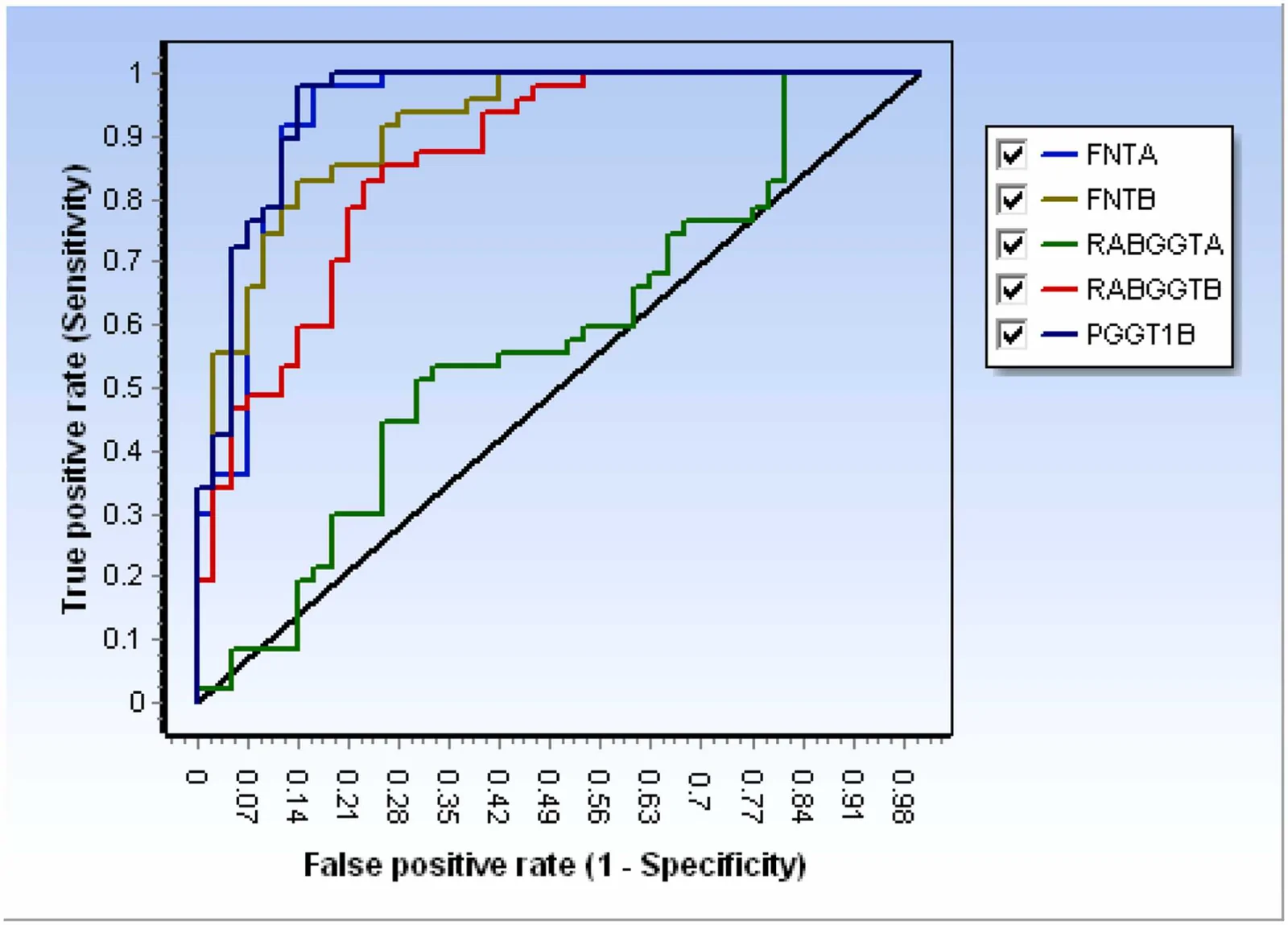
Receiver operating characteristic (ROC) curves for FNTA, FNTB, RABGGTA, RABGGTB, and PGGT1B in patients with Parkinson’s disease (PD) and healthy controls.

ROC analysis demonstrated strong discriminative performance for FNTA, FNTB, RABGGTB, and PGGT1B, whereas RABGGTA showed poor diagnostic utility. The highest AUC was observed for PGGT1B (AUC = 0.95, 95% CI 0.90–0.99), followed by FNTA (AUC = 0.93, 95% CI 0.88–0.99), FNTB (AUC = 0.91, 95% CI 0.88–0.97), and RABGGTB (AUC = 0.86, 95% CI 0.78–0.93); in contrast, RABGGTA yielded an AUC of 0.57 (95% CI 0.45–0.69). Sensitivity and specificity were highest for FNTA and PGGT1B, with favorable accuracy, PPV, and NPV values across these two genes (Table 5, Fig. 2). Table 6 further showed a clear classification shift toward the low-expression category in the PD group and toward the high-expression category in controls for FNTA, FNTB, RABGGTB, and PGGT1B, whereas RABGGTA displayed substantial overlap between groups.

**Table 5:** Diagnostic performance of ROC-derived cut-off values for FNTA, FNTB, RABGGTA, RABGGTB, and PGGT1B in differentiating patients with Parkinson’s disease (n = 43) from healthy controls (n = 47).

Gene	Cut-off ΔCt	Sensitivity (%)	Specificity (%)	Accuracy (%)	Positive Predictive Value	Negative Predictive Value	Area Under Curve (95% CI)
FNTA	< -6.704	83.7	97.8	91.1	97.3	86.8	0.93 (0.88–099)
FNTB	< -7.677	86.05	82.98	84.44	82.22	86.67	0.91 (0.88–0.97)
RABGGTA	< -9.193	69.7	51.06	60	56.6	64.86	0.57 (0.45–0.69)
RABGGTB	< -6.419	76.7	82.9	80	80.5	79.6	0.86 (0.78–0.93)
PGGT1B	< -7.886	86.05	97.87	92.22	97.37	88.46	0.95 (0.9–0.99)

**Table 6:** Classification of patients with Parkinson’s disease and healthy controls into high- and low-expression groups based on ROC-derived cut-off values for FNTA, FNTB, RABGGTA, RABGGTB, and PGGT1B (n, %).

Groups	FNTA High	FNTA Low	FNTB High	FNTB Low	RABGGTA High	RABGGTA Low	RABGGTB High	RABGGTB Low	PGGT1B High	PGGT1B Low
Healthy individuals	46 (97.9%)	1 (2.13%)	39 (83%)	8 (17%)	24 (51.1%)	23 (48.9%)	39 (83%)	8 (17%)	46 (97.9%)	1 (2.13%)
PD Patients	7 (16.3%)	36 (83.7%)	6 (14%)	37 (86%)	13 (30.2%)	30 (69.8%)	10 (23.3%)	33 (76.7%)	6 (14%)	37 (86%)

### Diagnostic performance of the final four-gene logistic regression model

**Fig. 3:**
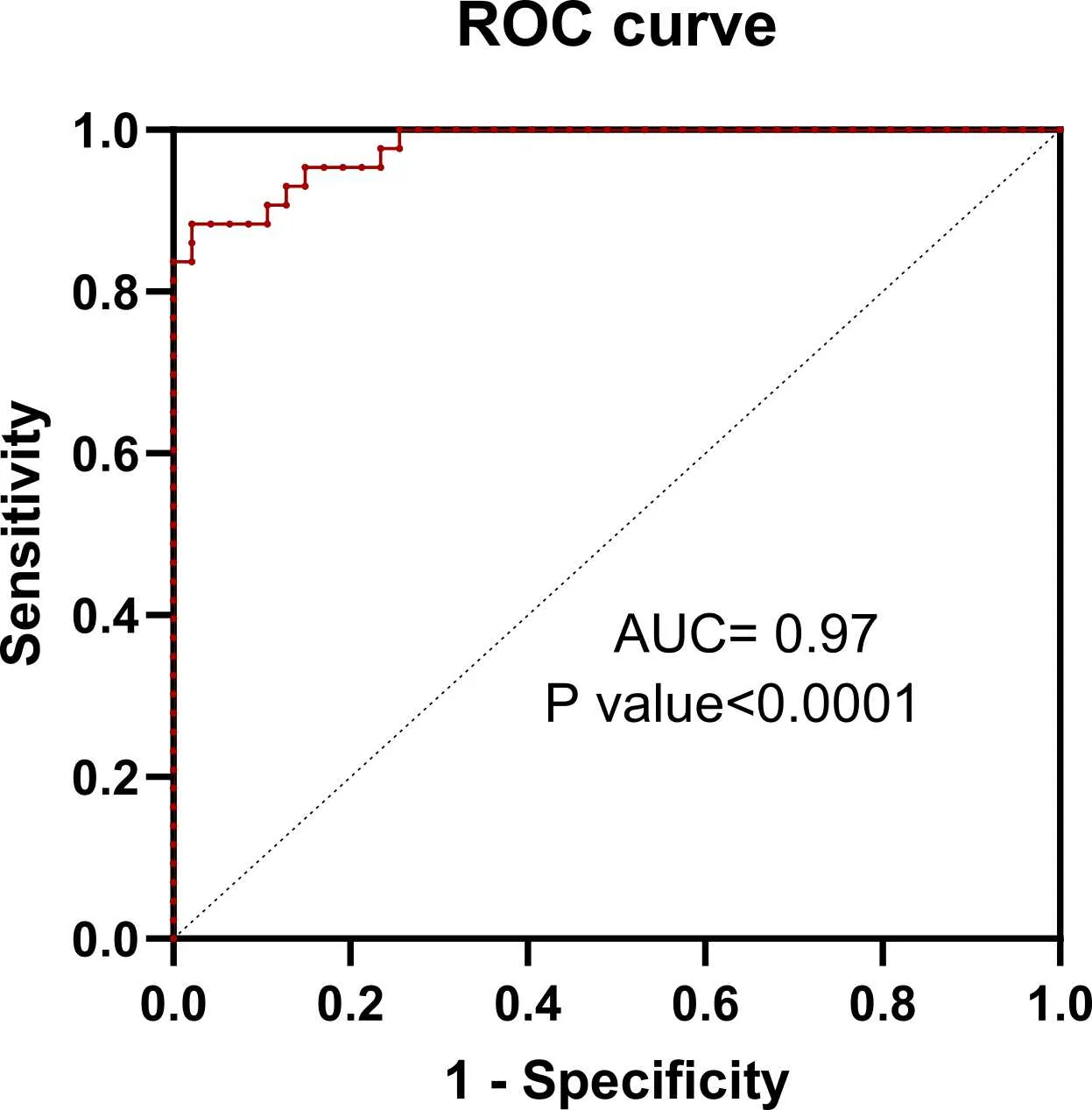
Receiver operating characteristic (ROC) curve of the multivariable logistic regression model combining FNTA, FNTB, RABGGTA, RABGGTB, and PGGT1B for discrimination between patients with Parkinson’s disease and healthy controls. The model showed good discriminatory performance, with an AUC of 0. 9787 (95% CI, 0. 9567–1; p < 0.0001).

Following exclusion of RABGGTA, the final multivariable logistic regression model comprising FNTA, FNTB, RABGGTB, and PGGT1B retained excellent and essentially unchanged discriminatory performance for distinguishing PD from controls (Fig. 3). The model yielded an AUC of 0.9787 (95% CI, 0.9567–1.000; *p* < 0.0001), with an overall classification accuracy of 90.0%. Correct classification was 91.49% for controls and 88.37% for patients, with corresponding PPV and NPV values of 90.48% and 89.58%, respectively. Model calibration was satisfactory, as indicated by a nonsignificant Hosmer–Lemeshow test (*p* = 0.8055), and model fit improved markedly relative to the intercept-only model, as reflected by the lower AICc (43.67 vs. 126.6). In the adjusted model, FNTA and PGGT1B remained inversely associated with disease status, RABGGTB showed a positive association, and FNTB did not retain statistical significance.

### Internal validation of the four-gene logistic regression model

The final logistic regression model incorporating FNTA, FNTB, RABGGTB, and PGGT1B showed an apparent AUC of 0.9787 (95% CI, 0.9567–1.000) and an overall classification accuracy of 90.0% when evaluated on the complete dataset. To assess potential optimism due to model fitting, stratified 5-fold cross-validation was subsequently performed. The cross-validated out-of-fold predictions yielded an AUC of 0.9466 and an overall classification accuracy of 88.9%. Thus, although the internally validated performance was modestly lower than the apparent performance, the model retained strong discriminatory ability after cross-validation. These findings should be considered evidence of internal robustness rather than external validation.

### Correlations between clinical variables and target-gene expression in PD group

Spearman’s rank correlation analysis was performed using the coded clinical variables (sex: 0 = female, 1 = male; disease duration: 0 = ≤1 year, 1 = >1 year; H&Y stage: 1–3). In the PD cohort, age was inversely correlated with MMSE score and positively correlated with FNTB expression. Disease duration was strongly correlated with H&Y stage and UPDRS score, and H&Y stage was inversely correlated with MMSE and positively correlated with UPDRS. Among the target genes, FNTB was the only transcript showing a significant association with age, whereas no significant correlations were observed between any gene and sex, disease duration, or UPDRS. RABGGTB showed a borderline positive association with H&Y stage, and FNTA showed a borderline inverse association with MMSE (Table 7).

**Table 7:** Spearman’s rank correlations between clinical variables and FNTA, FNTB, RABGGTA, RABGGTB, and PGGT1B expression in patients with Parkinson’s disease.

**Variable**	**Gender**	**Disease duration**	**Hoehn & Yahr stage**	**MMSE**	**UPDRS**	**FNTA**	**FNTB**	**RABGGTA**	**RABGGTB**	**PGGT1B**
**Age**	0.1 (0.51)	-0.29 (0.056)	0.005 (0.97)	-0.61*** **(<0.001)**	0.03 (0.85)	0.255 (0.084)	0.431** **(0.004)**	-0.06 (0.67)	0.22 (0.16)	0.18 (0.25)
**Gender**	—	-0.03 (0.86)	-0.08 (0.61)	-0.06 (0.68)	-0.09 0.53)	0 (1)	-0.05 (0.73)	0.01 (0.94)	-0.02 (0.91)	0.08 (0.61)
**Disease duration**	—	—	0.771*** **(<0.001)**	-0.21 (0.16)	0.57*** **(<0.001)**	-0.04 (0.81)	0.08 (0.6)	-0.004 (0.98)	0.18 **(**0.24)	0.13 (0.39)
**Hoehn & Yahr stage**	—	—	—	-0.46** **(<0.001)**	0.68*** **(<0.001)**	-0.014 (0.93)	0.017 (0.91)	-0.03 (0.84)	0.29 (0.057)	0.24 (0.11)
**MMSE**	—	—	—	—	-0.323* **(0.035)**	-0.29 (0.057)	-0.06 (0.67)	0.02 (0.91)	-0.24 (0.11)	-0.26 (0.09)
**UPDRS**	—	—	—	—	—	0.19 (0.21)	0.06 (0.7)	0.07 (0.66)	0.04 (0.76)	0.12 (0.42)

Values are Spearman’s rho (p-value).

**p < 0.001, *p < 0.01, p < 0.05.

## Discussion

In the present study, we identified a coordinated alteration in the peripheral expression of genes involved in the protein prenylation machinery in patients with PD. Specifically, *FNTA*, *FNTB*, *RABGGTB*, and *PGGT1B* were significantly downregulated in the PD group, whereas *RABGGTA* exhibited a less pronounced and statistically non-significant reduction. These findings were supported by multiple complementary analytical approaches, including differential expression analysis, multivariable regression, ROC curve analysis, and a combined multigene classification model. Together, these findings suggest that dysregulation of prenylation-related pathways may represent a previously underappreciated component of the molecular landscape of PD and may be detectable in peripheral blood.

The biological conceivability of these findings is supported by the essential role of protein prenylation in intracellular trafficking and neuronal homeostasis (Resh, 2006, Pisanti et al., 2022). Prenylation is an important post-translational modification that enables the membrane targeting and functional activity of numerous small GTPases, including members of the Rab family (Wang and Casey, 2016). These proteins regulate vesicle formation, intracellular transport, endosomal trafficking, autophagy, and lysosomal function, processes that are increasingly documented as crucial pathogenic mechanisms in PD (Bonet-Ponce and Cookson, 2019). Defects in vesicular trafficking and endolysosomal pathways have emerged as common downstream consequences of several PD-associated genes, such as *LRRK2*, *VPS35*, and *GBA1* (Usenko, 2025, Billingsley et al., 2018, Zavodszky et al., 2014). Consequently, alterations in genes encoding constituents of the prenylation machinery may have extensive implications for neuronal function and cellular homeostasis (Bonet-Ponce and Cookson, 2019, Jordan et al., 2022, Jiang et al., 2018).

Particularly relevant to the present findings is the growing evidence connecting Rab GTPases to PD pathogenesis (Bonet-Ponce and Cookson, 2019). Notably, pathogenic *LRRK2* variants has been found to increase phosphorylation of Rabs and subsequently decrease their affinity to regulatory proteins, thereby disrupting their normal interactions and altering intracellular trafficking dynamics (Steger et al., 2016). Furthermore, α-synuclein accumulation has been shown to disturb Rab-dependent membrane trafficking pathways, whereas restoration of Rab activity, particularly Rab1 and Rab11, ameliorates α-synuclein-mediated trafficking defects and cellular toxicity in experimental models of PD (Gitler et al., 2008, Cooper et al., 2006, Breda et al., 2015). Within this framework, down-regulation of FNTA, FNTB, RABGGTB, and PGGT1B may reflect a diminished capacity to maintain appropriate prenylation and membrane localization of Rab proteins, ultimately contributing to trafficking defects and impaired cellular homeostasis (Kiral et al., 2018). Although the present study was not designed to establish causality, the observed transcriptional changes are consistent with mechanisms that have already been implicated in PD biology.

Additionally, we reported coordinated involvement of genes encoding both farnesyltransferase and geranylgeranyltransferase subunits. *FNTA* and *FNTB* encode the α and β subunits of protein farnesyltransferase, whereas *PGGT1B* encodes the catalytic β subunit of geranylgeranyltransferase type I and *RABGGTB* encodes the catalytic β subunit of Rab geranylgeranyltransferase (Marchwicka et al., 2022). The simultaneous reduction of these transcripts suggests that the observed alterations are unlikely to reflect a gene-specific phenomenon and instead point toward a broader dysregulation of the prenylation apparatus. This interpretation is also supported by the correlation analyses, which demonstrated coordinated expression patterns among several of the studied genes. Notably, the correlation structure appeared stronger in healthy controls and somewhat attenuated in patients, suggesting that normal transcriptional coordination within this pathway may become disrupted in PD. Such findings are compatible with the concept of network-level dysregulation instead of isolated transcriptional abnormalities.

In this regard, it is important to mention that Rab geranylgeranylation is specifically mediated by the Rab geranylgeranyltransferase complex, including RABGGTB as its catalytic β subunit. FNTA and FNTB instead represent components of protein farnesyltransferase, while PGGT1B encodes the catalytic β subunit of geranylgeranyltransferase type I. The coordinated downregulation of *FNTA*, *FNTB*, *RABGGTB*, and *PGGT1B* is interpreted as a broader perturbation of the protein prenylation machinery rather than as evidence of a selective defect in Rab geranylgeranylation. Because protein prenylation regulates membrane association and the function of multiple small GTPases involved in intracellular trafficking, vesicular transport, and autophagy-related processes, coordinated alteration of different prenyltransferase systems may be biologically compatible with pathways implicated in PD. In addition, it is worth mentioning that the present study measured transcript abundance rather than enzyme activity or protein prenylation status; therefore, this interpretation remains hypothesis-generating and requires functional validation.

The relatively weak performance of *RABGGTA* deserves consideration. Although this gene participates in the Rab geranylgeranyltransferase complex (Marchwicka et al., 2022), its expression changes were modest and did not reach statistical significance. Moreover, its individual diagnostic performance was poor compared with the other genes, and its removal from the multivariable model produced virtually no change in overall classification performance. This observation suggests that *RABGGTA* contributes little independent information beyond that already captured by the remaining transcripts. Importantly, this finding should not necessarily be interpreted as evidence against a biological role for *RABGGTA* in PD. Instead, it may indicate a more tightly regulated expression of this transcript, less variable levels in peripheral blood, or minimal impact of disease-related mechanisms on its expression compared with other components of the prenylation machinery.

One of the most notable findings of the current study was the strong discriminatory ability of the combined gene-expression model. While individual genes showed variable degrees of diagnostic performance, the integrated model achieved an AUC approaching 0.98 and maintained high classification accuracy even after exclusion of RABGGTA. These findings demonstrate the value of pathway-based biomarker strategies. Neurodegenerative diseases are essentially complex and are unlikely to be sufficiently represented by a single molecular marker. Instead, coordinated alterations across multiple biologically related genes may provide a stronger reflection of disease-associated processes (Koníčková et al., 2022, Cheslow et al., 2024). The excellent performance of the combined model therefore supports the concept that peripheral dysregulation of the prenylation pathway may serve as a composite molecular signature of PD.

Nevertheless, the diagnostic performance observed in the present study should be interpreted with appropriate caution. Biomarker studies frequently report excellent performance in discovery cohorts, whereas substantially lower accuracy is often observed during external validation. Consequently, the current results should be regarded as evidence of strong internal validity rather than proof of immediate clinical applicability. Independent replication in larger and geographically distinct cohorts will be required prior to any conclusions regarding clinical utility. Moreover, longitudinal studies will be necessary to determine whether alterations in prenylation-related gene expression are stable throughout disease progression or change in response to treatment and disease severity.

The relationship between the observed transcriptional changes and disease mechanisms also warrants further investigation. Recent experimental studies have suggested that modulation of prenylation pathways may influence autophagic-lysosomal function, a process that is intensely disrupted in neurodegenerative diseases (Shao et al., 2024). Farnesyltransferase inhibition has been reported to rescue defects in autophagic-lysosomal fusion in patient-derived neuronal models, highlighting a direct mechanistic connection between prenylation and pathways implicated in neurodegeneration (Pitcairn et al., 2023). In this context, the reduced expression of prenylation-related genes observed in our study may represent either a maladaptive pathogenic event or a compensatory response to ongoing cellular stress. Distinguishing between these possibilities will require functional studies examining enzyme activity, protein prenylation status, and downstream trafficking defects in both peripheral cells and disease-relevant neuronal systems.

Several strengths of the present study should be accredited. Gene selection was guided by a coherent biological hypothesis centered on the prenylation pathway rather than by exploratory transcriptome-wide screening. Multiple complementary statistical approaches yielded convergent results, enhancing confidence in the strength of the findings. Besides, the use of peripheral blood samples increases the translational potential of the identified signature, as blood-based biomarkers are considerably more feasible for routine clinical application than biomarkers requiring invasive sampling procedures.

At the same time, several limitations should be considered. The study employed a cross-sectional design and was conducted in a single cohort with a relatively modest sample size. Gene expression was measured in peripheral blood rather than in brain tissue, and therefore the extent to which these alterations directly reflect central nervous system pathology remains uncertain. Medication use may also have influenced transcript levels, and differences in blood-cell composition could have contributed to some of the observed expression changes. Medication exposure represents an important potential confounder in the present study. Most patients received L-DOPA-containing regimens; however, individual L-DOPA dose information was not systematically available, precluding reliable dose–response and medication-adjusted analyses. Therefore, the present findings cannot completely disentangle disease-associated transcriptional changes from potential treatment-related effects. In addition, although the handling of non-detect values followed a predefined and conservative strategy, the potential influence of this approach on expression estimates cannot be completely excluded. Finally, the absence of external validation represents the most important limitation of the current work. Finally, because detailed differential blood-cell composition data were not systematically available, the potential contribution of differences in leukocyte composition to the observed transcriptomic changes could not be directly assessed and therefore remains a possible source of residual confounding.

In conclusion, the present study demonstrates significant downregulation of multiple genes encoding key components of the protein prenylation machinery in the peripheral blood of patients with PD. The convergence of differential expression analyses, correlation patterns, and multigene classification modeling supports the existence of a coordinated prenylation-related transcriptional signature in PD. These findings provide further evidence linking Rab-associated trafficking pathways and prenylation biology to PD and suggest that peripheral expression profiles of prenylation-related genes may represent promising candidates for future biomarker development and mechanistic investigation. However, we emphasize that the findings represent preliminary evidence supporting further biomarker development and require replication in independent and larger cohorts before clinical utility can be assessed.

## CRediT authorship contribution statement

**Mohammad Jalal Tabatabaie:** Investigation, Data curation. **Solat Eslami:** Formal analysis. **Atefe Abak:** Methodology. **Soudeh Ghafouri-Fard:** Writing – review & editing, Writing – original draft, Supervision.

## Ethics declaration

Written informed consent to take part in the study and to publish the article has been obtained from all participants or their legal representatives. The privacy rights of participants have been observed.

This study included organ or tissue donors. This study includes human biological material and consent was obtained by donors, or their next of kin or legal representatives, for use in this study and for publication of the article. The samples used in this research were not sourced from executed prisoners or prisoners of conscience.

This study was performed in compliance with relevant laws, regulatory frameworks and guidelines where the research took place. This study was approved by the Shahid Beheshti University of Medical Sciences. (Approval No. IR.SBMU.MSP.REC.1404.443).

## Ethics approval and consent to participate

All procedures were in accordance with the ethical standards of the institutional and national research committee and with the 1964 Helsinki declaration and its later amendments or comparable ethical standards. Informed consent forms were obtained from all study participants. The study protocol was approved by the ethical committee of Shahid Beheshti University of Medical Sciences (IR.SBMU.MSP.REC.1404.443).

## Consent of publication

Not applicable.

## Funding

This study was supported by 10.13039/501100005851Shahid Beheshti University of Medical Sciences.

## Competing Interest

The authors declare they have no conflict of interest.

## Data Availability

All data generated or analyzed during this study are included in this published article.
